# Rapamycin increases oxidative stress response gene expression in adult stem cells

**DOI:** 10.18632/aging.100451

**Published:** 2012-04-18

**Authors:** Amber E. Kofman, Margeaux R. McGraw, Christopher J. Payne

**Affiliations:** ^1^ Human Molecular Genetics Program, Children's Memorial Research Center, Chicago, IL 60614, USA; ^2^ Departments of Pediatrics and Obstetrics and Gynecology, Northwestern University Feinberg School of Medicine, Chicago, IL 60611, USA

**Keywords:** adult stem cells, testis, antioxidant, rapamycin, aging

## Abstract

Balancing quiescence with proliferation is of paramount importance for adult stem cells in order to avoid hyperproliferation and cell depletion. In some models, stem cell exhaustion may be reversed with the drug rapamycin, which was shown can suppress cellular senescence *in vitro* and extend lifespan in animals. We hypothesized that rapamycin increases the expression of oxidative stress response genes in adult stem cells, and that these gene activities diminish with age. To test our hypothesis, we exposed mice to rapamycin and then examined the transcriptome of their spermatogonial stem cells (SSCs). Gene expression microarray analysis revealed that numerous oxidative stress response genes were upregulated upon rapamycin treatment, including superoxide dismutase 1, glutathione reductase, and delta-aminolevulinate dehydratase. When we examined the expression of these genes in 55-week-old wild type SSCs, their levels were significantly reduced compared to 3-week-old SSCs, suggesting that their downregulation is coincident with the aging process in adult stem cells. We conclude that rapamycin-induced stimulation of oxidative stress response genes may promote cellular longevity in SSCs, while a decline in gene expression in aged stem cells could reflect the SSCs' diminished potential to alleviate oxidative stress, a hallmark of aging.

## INTRODUCTION

Cell senescence may contribute to adult stem cell exhaustion, compromising the maintenance of cell lineages within the body [1]. Recent evidence suggests that the cumulative exposure to reactive oxygen species (ROS) and DNA damage can lead to the decline of adult stem cells both in population and in regenerative capacity. For example, hematopoietic stem cells (HSCs) from mice lacking forkhead box O (FOXO) family transcription factors exhibit higher levels of ROS, accompanied by short-term hyperproliferation that is then followed by increased apoptosis that depletes the HSC pool [2, 3]. Epithelial stem cells in the epidermis (ESCs) that are engineered to constitutively transduce wingless-related MMTV integration site (WNT) signals in mice rapidly divide in the short term, but then undergo cell senescence and disappear from the ESC compartment [4]. Neural stem cells (NSCs), meanwhile, decline in number and function within the subventricular zone of lateral ventricles in the aging mouse brain due to genomic instability and upregulated cyclin-dependent kinase inhibitor 2a (*Cdkn2a*; *p16^Ink4a^*), which activates DNA-damage response pathways that induce apoptosis or senescence [5, 6]. Spermatogonial stem cells (SSCs) exhibit a loss of regenerative ability during aging *in vivo* and *in vitro*, with the downregulation of several genes important for self-renewal [7-9, 10].

Altered expression of the mammalian target of rapamycin complex 1 (mTORC1), a key regulator of cell metabolism and a kinase whose downstream activity is associated with phosphatidylinositol 3-kinase (PI3-K) signaling pathways, significantly alters the fate of adult stem cells. Studies in mice have revealed that hyperactive signaling through mTORC1 depletes HSCs, ESCs, and SSCs from their respective compartments [4, 11, 12]. This stem cell loss is rescued upon exposure to the drug rapamycin, with wild type SSCs undergoing active expansion *in vivo* [12]. Rapamycin specifically inhibits mTORC1 and has been shown to increase the lifespan of organisms, including worms, flies, and aging mice [13-17]. Recent evidence demonstrated that rapamycin decreases mammalian cell senescence and delays spontaneous tumor development in mice at older ages [18, 19]. Insulin signaling and insulin-like growth factor 1 receptor activation, meanwhile, are known to modulate the levels of enzymes regulating numerous cellular processes. When wild type mice or cultured endothelial cells are exposed to high levels of glucose to establish diabetes-associated conditions, the transcriptional activity of superoxide dismutase 1 (*Sod1*) and the enzymatic activity of delta-aminolevulinate dehydratase (ALAD) are significantly lower than in controls [20-22]. Levels of glutathione and the enzyme glutathione reductase (GSR) are depleted in apoprotein E-deficient mutant mice [23]. As putative biomarkers for oxidative stress, *Sod1*, *Gsr*, and *Alad* transcript levels might also be expected to be altered in adult stem cells upon elevated mTORC1 activity or during the aging process.

Here, using mouse SSCs as an *in vivo* model system for studying adult stem cell maintenance and gene regulation downstream of mTORC1, we investigated the effect of rapamycin on the SSC transcriptome. We found that mTORC1 inhibition not only upregulates key genes important for SSC self-renewal, but also elevates transcript levels of oxidative stress response genes and downregulates genes associated with growth and metabolism. When aged SSCs were examined for *Sod1*,*Gsr*, and *Alad*, these transcript levels were significantly reduced when compared with those of younger SSCs. Our results implicate the aging process and mTORC1 in downregulating oxidative stress response genes in adult stem cells.

## RESULTS

### Magnetic-activated cell sorting enriches undifferentiated male germ cells from rapamycin-treated mice

**Figure 1:**
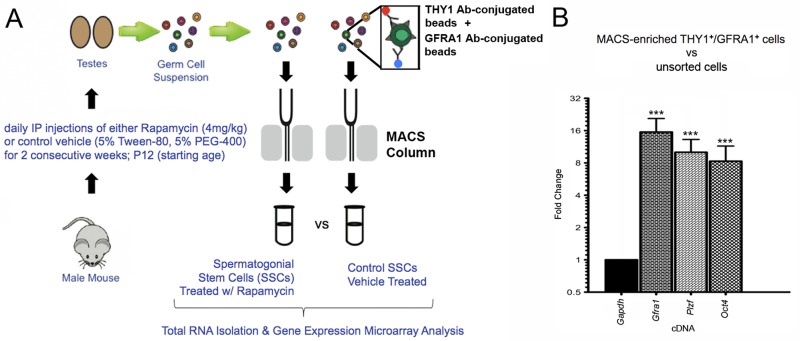
Magnetic-activated cell sorting (MACS) significantly enriches for SSCs isolated from rapamycin- or control-treated mice Male animals were chronically administered rapamycin or control vehicle for 2 weeks, followed by MACS selection for undifferentiated germ cells. (**A**) Schematic diagram depicting the experimental design. Isolated testes were enzymatically digested to single cell suspensions containing germ cells (multi-colored circles). Incubation with magnetic beads conjugated to antibodies that recognize SSC surface proteins THY1 and GFRA1 was followed by the passage of the samples through separation columns attached to a magnet (gray bars). THY1+ and GFRA1+ cells were ultimately flushed out for cell culture or RNA isolation. (**B**) Quantitative RT-PCR was performed on THY1+/GFRA1+ cells enriched by MACS from age-matched wild type (non-injected) mice. When compared to the endogenous control *Gapdh* (assigned a relative value of “1”), the fold-changes in expression of SSC markers *Gfra1*, *Plzf*, and *Oct4* were all significantly elevated in the MACS-selected cells versus unsorted testicular cells (16-fold, 10-fold, and 8-fold, respectively). Data represent mean values +/− SEM from three biological replicates. Student's t-test was performed to assess significance between each SSC marker and the endogenous control; ***p<0.001.

To examine the effects of mTORC1 inhibition on SSC gene expression, we first implemented an established regimen in which juvenile male mice were administered intraperitoneal injections of rapamycin or control vehicle daily for two weeks (Figure 1A) [4, 12, 24]. Following these treatments, single cell suspensions of germ cells were prepared from isolated testes and subjected to magnetic-activated cell sorting (MACS). This procedure enriches the undifferentiated germ cell fraction, which represents the adult SSC population (Figure 1A) [25-27]. RNA from cells double-positive for the SSC surface markers thymus cell antigen 1, theta (THY1) and glial cell line-derived neurotrophic factor family receptor alpha 1 (GFRA1) was isolated for gene expression microarray analysis and quantitative reverse transcription-polymerase chain reaction (qRT-PCR) validation. In order to verify that our MACS selection strategy was successfully enriching undifferentiated male germ cells, we performed qRT-PCR on MACS-enriched THY1^+^/GFRA1^+^ cells from non-injected mice. When compared to unsorted germ cells, the MACS-enriched cells exhibited a 16-fold increase in *Gfra1* transcripts, as well as 10-fold and 8-fold increases, respectively, in two additional SSC transcripts, zinc finger and BTB domain containing 16 (*Zbtb16*; referred to here as *Plzf*) and POU domain, class 5, transcription factor 1 (*Pou5f1*; referred to here as *Oct4*) (Figure 1B). MACS-enriched THY1^+^ and GFRA1^+^ germ cells have previously been shown to exhibit significantly increased stem cell activity over unsorted cells through transplantation assays [25, 28]. Thus, our experimental design favorably selected for SSCs and allowed for the examination of rapamycin-induced alterations of SSC gene activity.

### Rapamycin administration inhibits testis growth but expands the number of undifferentiated germ cells

**Figure 2:**
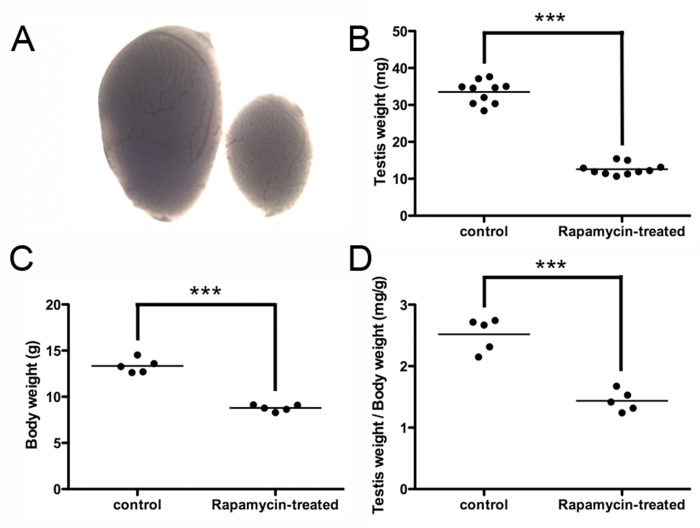
Rapamycin inhibits testis growth and reduces body weight (**A**) Testes from mice chronically treated with rapamycin for 2 weeks (days 12 through 25) were significantly smaller in size. Representative images of a rapamycin-exposed testis (right) and a control vehicle-exposed testis (left). (**B**) Rapamycin-exposed mouse testes exhibited significantly diminished weights (12.60 ± 0.492 mg vs 33.52 ± 0.968 mg average testis weight; N=10, ***p<0.001). (**C**) Body weights of rapamycin-exposed mice were significantly reduced (8.783 ± 0.155 g vs 13.35 ± 0.345 g average body weight; N=5, ***p<0.001). (**D**) When controlled for body weight, rapamycin-exposed testes exhibited significantly lower values (1.44 mg/g body weight vs. 2.52 mg/g body weight; N=5, ***p<0.001).

**Figure 3:**
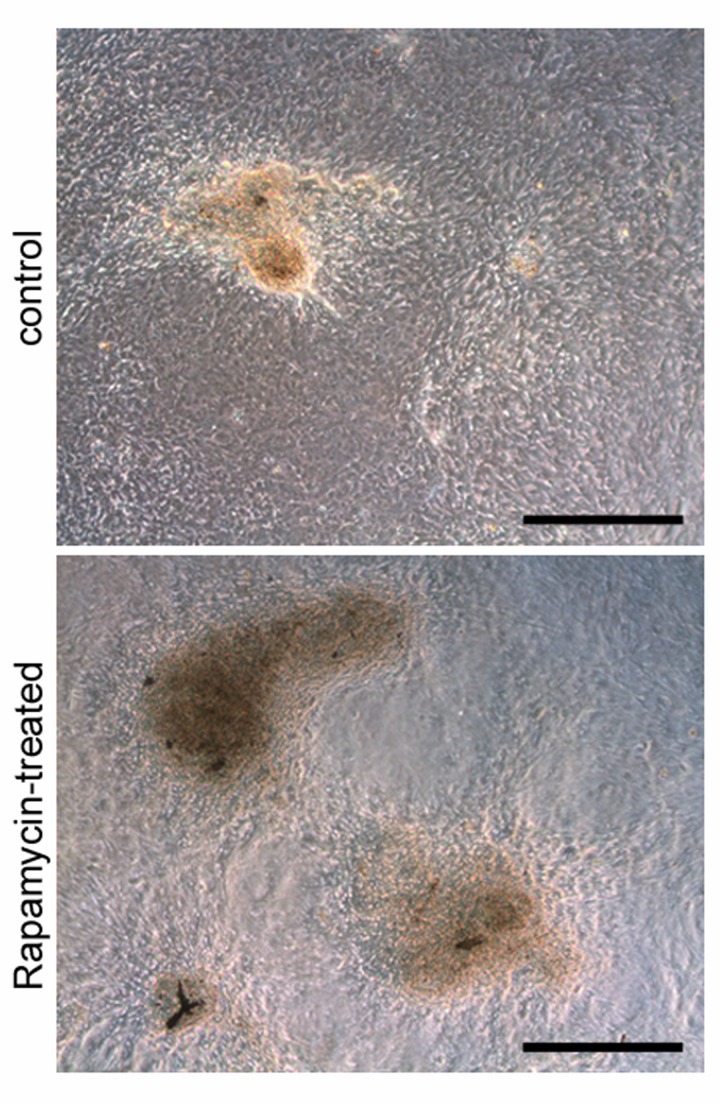
Rapamycin expands SSC colonies in culture MACS-enriched THY1+/GFRA1+ cells cultured *in vitro* for two weeks from rapamycin-treated mice formed more numerous and larger-sized colonies (bottom) than the control SSCs (top). Scale bars = 200 μm.

Testicular weights and body weights of rapamycin-treated mice were both significantly reduced compared to those of control vehicle-treated mice (12.60 ± 0.492 mg vs 33.52 ± 0.968 mg average testis weight; 8.783 ± 0.155 g vs 13.35 ± 0.345 g average body weight; N=5), reflecting the effects of rapamycin on tissue growth (Figure 2 A-D). Administration of rapamycin or control vehicle began on postnatal day (P)12 and ended on P25, a period of mouse development in which the testis generates the first sets of meiotic and post-meiotic germ cells [29]. Equivalent numbers of terminally differentiated Sertoli cells, but fewer mitotic progenitor Leydig cells, were observed between rapamycin- and control vehicle-treated mice (data not shown). Rapamycin had previously been shown to inhibit the proliferation of differentiating germ cells through PI3-K signaling [30]. Conversely, mTORC1 inhibition by rapamycin expands the number of SSCs through glial cell line-derived neurotrophic factor (GDNF) signaling [12]. When we placed SSCs from individual mice into culture, stem cells from the rapamycin-treated mice formed more numerous and larger-sized colonies than the control SSCs during the first two weeks after plating (Figure 3). These results demonstrated that chronic rapamycin exposure selected for adult SSCs within the germ cell population of the juvenile testis. From this, we wondered whether the selection involved transcriptional networks independent of the GDNF signaling pathway.

### Identification and validation of a rapamycin-induced SSC transcriptome signature

**Figure 4:**
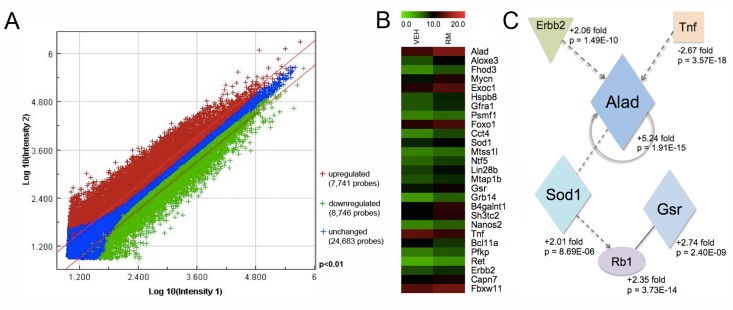
Transcriptional profiling of rapamycin-exposed SSCs reveals an upregulation of oxidative stress response genes (**A**) Double-log scatter plot to visualize the signal intensities of all oligo probes on the Agilent Whole Mouse Genome oligonucleotide microarray 4x44K platform that were upregulated (red crosses), downregulated (green crosses), or unchanged (blue crosses). Filter conditions applied to the scatter plot: >2-fold change and p-value <0.01. (**B**) Heat map of genes within a cluster containing *Gfra1* that were significantly altered in THY1+/GFRA1+ cells in response to rapamycin exposure. Horizontal stripes represent genes and columns show the treatment conditions (VEH = control vehicle; RM = rapamycin). Log2-fold changes of gene ratios are color coded as shown in the top horizontal stripe, from a relative low value of 0.0 (green) to a high of 20.0 (red). (**C**) Potential relationships among the antioxidant gene products of *Sod1*, *Gsr*, and *Alad* in rapamycin-exposed SSCs, illustrated here as a schematic adapted from Ingenuity Pathways Analysis^®^ (IPA). Parallelograms represent enzymes, ovals represent transcription factors, squares represent cytokines, and inverted triangles represent cell receptors. Solid lines indicate direct binding (only) between gene products, while dashed lines represent indirect associations. Arrows indicate that the first product acts upon the second product. A circular solid line with arrow represents direct auto regulation. Fold changes and p-values are listed next to the gene products.

**Figure 5:**
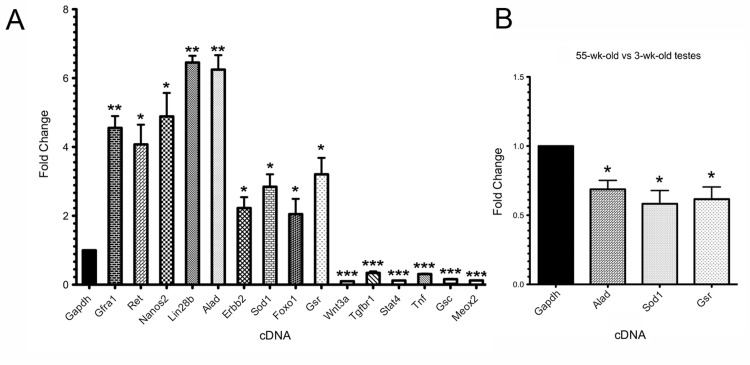
Microarray validation and determination that genes encoding antioxidants are downregulated in aging SSCs (**A**) Quantitative RT-PCR validation of the differential gene expression of 15 selected transcripts observed in the microarray analysis of rapamycin-exposed SSCs (THY1+/GFRA1+ cells enriched by MACS). Nine upregulated genes, *Gfra1*, *Ret*, *Lin28b*, *Nanos2*, *Foxo1*, *Alad*, *Sod1*, *Gsr*, *Erbb2*, and six downregulated genes, *Wnt3a*, *Tgfbr1*, *Stat4*, *Tnf*, *Gsc*, *Meox2*, were compared to endogenous control *Gapdh*(assigned a relative value of “1”). (**B**) Quantitative RT-PCR compared THY1+/GFRA1+ cells enriched by MACS from 55-week-old wild type males to MACS-enriched cells from 3-week-old controls. When compared to endogenous control *Gapdh*, the fold-changes in expression of *Alad*, *Sod1*, and *Gsr* were all significantly diminished in the aged SSCs versus the young SSCs (1.46-, 1.72-, and 1.62-fold, respectively).Data represent mean values +/− SEM from three biological replicates. Student's t-test was performed to individually assess significance between each transcript and endogenous control *Gapdh*; *p<0.05, **p<0.01, ***p<0.001.

To identify differentially expressed genes in rapamycin-exposed mouse SSCs, we used the Agilent Whole Mouse Genome oligonucleotide microarray 4x44K platform. Approximately 40% of the SSC transcriptome was significantly altered in expression levels, as measured by >2-fold change and p-value <0.01 (Figure 4A). A total of 7,741 oligo probes were upregulated, while 8,746 probes were downregulated, corresponding to an enhancement of 4,617 transcripts (unique Entrez gene IDs) and a reduction of 5,360 transcripts (Figure 4A, Supplemental Table 1). For data analysis, we used the non-hierarchical clustering method AutoSOME and generated a heat map of the cluster that specifically contained *Gfra1* (Figure 4B) [31]. The *Gfra1* transcript had previously been shown to be upregulated in SSCs following rapamycin exposure, and it exhibited a 3.13-fold enhancement in expression here (Table 1)[12]. Within the *Gfra1* cluster were additional SSC self-renewal-associated genes (*Ret*, *Lin28b*,*Nanos2*, *Foxo1*), all of which were significantly upregulated in our rapamycin-exposed SSCs (Figure 4B, Table 1). In addition to these genes important for SSC maintenance, the *Gfra1* cluster contained several oxidative stress response genes that were also significantly upregulated with rapamycin, including *Alad*, *Sod1*, and *Gsr* (Figure 4B, Table 1). In contrast, genes important for signal transduction in growth and metabolism (*Wnt3a*, *Wnt2*, *Stat4*, *Tgfbr1-3*) were significantly downregulated in our rapamycin-exposed SSCs (Table 1). To integrate these SSC transcriptome data into biological pathways, we used the knowledge-based database Ingenuity Pathway Analysis^®^ (IPA). The top five canonical pathways, ranked by p-value and ratio of the number of genes (up/down) per category, are listed in Table 2. These include free radical scavenging, phosphatase and tensin homolog (PTEN) signaling, and nuclear factor (erythroid-derived 2)-related factor 2 (NRF2)-mediated oxidative stress response (Table 2). IPA identified several potential interactions among the oxidative stress response genes in our *Gfra1* cluster, with ALAD serving as a nodal point to connect tumor necrosis factor (TNF) and erythroblastic leukemia viral oncogene homolog 2, neuro/glioblastoma derived oncogene homolog (ERBB2) with SOD1, GSR, and retinoblastoma protein (RB1) (Figure 4C). We validated the differential regulation of 15 selected transcripts (9 upregulated: *Gfra1*, *Ret*, *Lin28b*, *Nanos2*,*Foxo1*, *Alad*, *Sod1*, *Gsr*, *Erbb2*; 6 downregulated: *Wnt3a*, *Tgfbr1*, *Stat4*, *Tnf*, *Gsc*,*Meox2*) from the gene expression microarray using qRT-PCR (Figure 5A). All genes exhibited significant changes in expression as predicted from the microarray data.

**Table 1:** Selected differentially expressed genes from the oligo microarray (rapamycin-exposed SSCs vs controls)

Category		Fold Δ		p value
		UP	DOWN	
SSC self-renewal				
	*Gfra1*	3.13411		4.35E-10
	*Ret*	2.71904		4.69E-06
	*Lin28b*	5.77946		0.00615
	*Nanos 2*	3.00648		0.00007
	*Foxo1*	2.3856		8.50E-08
Signal transduction				
	*Wnt3a*		8.76346	1.98E-07
	*Wnt2*		3.28504	4.55E-10
	*Stat4*		7.53744	8.22E-18
	*Tgfbr1*		2.52916	3.41E-08
	*Tgfbr2*		2.41742	6.11E-08
	*Tgfbr3*		2.98383	8.50E-06
Oxidative stress response				
	*Sod1*	2.01295		8.69E-06
	*Gsr*	2.74239		2.40E-09
	*Alad*	5.23738		1.91E-15
	*Gstm6*	6.59408		5.00E-17
	*Gstm7*	7.34355		1.15E-17
	*Brca1*	2.91830		0.00183
	*Nfe2l1*	2.21224		5.94E-07
	*Glrx2*	2.75994		0.00039
Other				
	*Erbb2*	2.06434		1.49E-10
	*Rb1*	2.35478		3.73E-14
	*Tnf*		2.67337	3.57E-18
	*Gsc*		5.13706	1.74E-13
	*Meox2*		7.68215	3.37E-06

**Table 2:** Canonical pathways identified by Ingenuity Pathway Analysis^®^

Category (up/down genes)	p value	ratio (# genes/category)
Free radical scavenging	5.51E-04	26/57 (0.456)
PTEN signaling	2.96E-04	48/114 (0.421)
Growth Hormone signaling	5.50E-03	30/69 (0.435)
NF-κB signaling	2.67E-03	64/165 (0.388)
NRF2-mediated oxidative stress response	1.47E-02	64/180 (0.356)

### Oxidative stress response genes upregulated by rapamycin are downregulated in aging SSCs

Given that rapamycin has been shown to increase lifespan in aging mice [13, 19], and that here it significantly enhanced the expression of *Alad*, *Sod1*, and *Gsr* in juvenile SSCs, we next asked whether the levels of these three oxidative stress response transcripts were diminished in the SSCs isolated from older versus younger wild type mice. When SSCs from 55-week-old males were compared to SSCs from 3-week-old males, the relative gene expression values for *Alad*, *Sod1*, and *Gsr* were all decreased (1.46-, 1.72-, and 1.62-fold, respectively; p<0.05) (Figure 5B). Morphologically, the SSCs from the two ages of mice were indistinguishable, although fewer SSCs were obtained from the older testes than from the younger testes (data not shown). These data suggest that as SSCs age *in vivo*, the aging process correlates with a downregulation in the expression of genes that respond to oxidative stress.

## DISCUSSION

The maintenance of adult stem cells, including SSCs, is critical to ensure the continuous production of differentiated cells within that lineage as organisms age. Mouse SSC self-renewal is promoted through GDNF signaling and mTORC1 antagonism by the PLZF-mediated activation of *Redd1* [12]. Chronic exposure of mouse testes to rapamycin expands the SSC pool *in vivo* and increases *Gfra1* and *Ret* expression [12]. The present study demonstrated that along with *Gfra1* and *Ret*, additional SSC self-renewal genes (*Lin28b*, *Nanos 2*, *Foxo1*) and oxidative stress response genes (*Alad*, *Sod1*, *Gsr*) are upregulated in rapamycin-exposed SSCs. LIN28B suppresses microRNA biogenesis through interactions with the *let-7* precursor, and is enriched in undifferentiated germ cells within the testis [32]. The functional role of LIN28B in SSCs is not yet clear, but the protein exhibits a striking temporal co-expression in germ cells with PLZF, suggesting a possible regulatory association with this transcription factor (unpublished observations). NANOS2 is an RNA-binding protein that acts downstream of GFRA1 to promote SSC self-renewal, and is required for stem cell maintenance [33, 34]. FOXO1, a transcription factor, regulates the expression of *Ret* and other genes in SSCs and is required for their homeostasis [35]. Collectively, these findings identify a transcriptional network that is enhanced when mTORC1 is inhibited by rapamycin.

Elevated levels of transcripts encoding antioxidant enzymes in our rapamycin-exposed SSCs suggest the possibility that the mitigation of ROS and DNA damage could facilitate adult stem cell expansion and that it might be counterbalanced by mTORC1 activity. An association between*Sod1* and the inhibition of mTORC1 by rapamycin was recently demonstrated in yeast, and enhanced GSR activity was observed in rapamycin-treated human corneal endothelial cells exposed to tert-butyl hydroperoxide [36, 37]. SOD1 binds to copper and zinc ions within the cytoplasm and mitochondrial intermembrane, and is one of three superoxide dismutase enzymes that destroy free superoxide radicals. GSR reduces glutathione disulfide, GSSG, into the sulfhydryl form, GSH. To our knowledge, no relationship between *Alad* and mTORC1 had previously been identified. ALAD activity is a biomarker for oxidative stress in human bone marrow transplant recipients, as well as for lead toxicity in human populations [38, 39]. ALAD catalyzes the second step in porphyrin and heme biosynthesis, condensing two molecules of delta-aminolevulinate to form porphobilinogen. We speculate that rapamycin might upregulate antioxidants in SSCs at the transcriptional level to promote cellular longevity, with the concomitant inhibition of metabolic events to reduce ROS and oxidative damage. Indeed, the top canonical pathways predicted by IPA to be differentially regulated upon rapamycin exposure involve biological processes known to associate with metabolism and its consequences: growth hormone signaling, NF-κB signaling, PTEN signaling, free radical scavenging, and the NRF2-mediated oxidative stress response. PTEN is a negative regulator of mTORC1, while NF-κB is activated downstream of mTORC1 to modulate specific sets of genes [11, 40]. Additional processes activated downstream of mTORC1 include ribosome biogenesis and protein synthesis, cellular events that, when inhibited, can extend lifespan (reviewed in [41]). Interestingly, an association between NRF2 and mTORC1 was recently identified using *in vitro* and *in vivo* models of lung cancer [42].

Many types of tissue-specific adult stem cells share common defects as they age within their respective niches. Aging mouse SSCs (>1 year) exhibit functional deficiencies, showing reduced proliferation *in vitro* and diminished colonization ability *in vivo* [7]. Kokkinaki et al. [8] compared 8-month-old SSCs to 1-week-old SSCs and identified a number of genes downregulated in the older cells, including membrane-spanning 4-domains, subfamily A, member 7 (*Ms4a7*), which mediates cell proliferation [8, 43]. *Drosophila*male germline stem cells (GSCs) experience a decline in self-renewal factors and a misorientation of their centrosomes as they age, contributing to fewer cell divisions [44, 45]. Aging mouse HSCs exhibit reduced numbers and diminished function, while aging melanocyte stem cells incur DNA damage and reflect inappropriate differentiation [46, 47]. Evidence of a decline in oxidative stress response gene activity during the aging process is contradictory. Aging rat bone marrow cells show no differences in ALAD levels in one study, while blood from aging rats contain progressively diminished ALAD in a different report [48, 49]. Mouse ovarian cells (2-, 6-, 9-, and 12-months) upregulate glutathione peroxidase 1 (*Gpx1*), but downregulate glutaredoxin 1 (*Glrx1*), glutathione S-transferase mu 2 (*Gstm2*), peroxiredoxin 3 (*Prdx3*), and thioredoxin 2 (*Txn2*) as they age [50]. *GPX1* expression and activity decrease, however, in aging human endothelial progenitor cells [51]. Overexpression of *SOD1* in aging *Drosophila* female GSCs and mouse NSCs increases their cellular proliferation and prolongs their maintenance, while endogenous SOD1 activity diminishes nearly 1.5-fold in aged rat gastrocnemius muscle (7-month-old versus 30-month-old) [52-54].

Here we have shown that aged mouse SSCs (>1-year-old) exhibit significantly reduced levels of *Sod1*, *Gsr*, and *Alad* transcripts when compared to young SSCs (<1-month-old). This reduction in gene expression likely affects the function of these stem cells within their niche, warranting further studies to examine the physiological consequences of this downregulation. While ROS accumulation is only one of many potential cellular mechanisms associated with aging that have yet to be fully investigated, the lower expression levels of oxidative stress response genes observed in aging SSCs could reinforce the diminished capacity for self-renewal in these stem cells. Such a process might, in turn, be reversed through rapamycin administration as a possible therapeutic endeavor.

## METHODS

### Animals

Male FVB mice aged 12-days-old through 26-days-old were administered daily intraperitoneal (IP) injections of rapamycin (4mg/kg body weight) or control vehicle (5% Tween-80, 5% PEG-400), beginning at postnatal day (P)12. Mice were euthanized at P26 and their testes were isolated for germ cell enrichment. Untreated wild type male FVB mice aged 3-weeks-old and 55-weeks-old were also euthanized for experimental analysis. All procedures and care of animals were carried out according to the Children's Memorial Research Center Animal Care and Use Committee.

### Isolation of mouse testicular germ cells

Testes were decapsulated and briefly minced in ice-cold 1:1 Dulbecco's Modified Eagle Medium-Ham's F-12 Medium. An initial enzymatic digestion using collagenase IV (1mg/ml) and DNase I (2mg/ml) at 37°C for 30 min. was administered to remove interstitial Leydig cells and peritubular myoid cells from the seminiferous tubules. A second enzymatic digestion using collagenase IV (1 mg/ml), DNase I (2mg/ml), hyaluronidase (1.5mg/ml), and trypsin (1mg/ml) at 37°C for 30 min. was administered to isolate germ cells and Sertoli cells from the remaining tissue. A final suspension of single cells was prepared in ice-cold PBS containing 0.5% BSA and 2mM EDTA (MACS Buffer) for subsequent spermatogonial stem cell enrichment.

### Enrichment of SSCs using MACS separation

The single cell suspension containing germ cells in 80 μl MACS Buffer was first incubated with 20 μl rabbit anti-GFRA1 antibodies (Santa Cruz Biotechnology, CA) at 4°C for 20 min. with rotation. After washes, a second incubation of cells in 80 μl MACS Buffer with 10 μl goat anti-rabbit antibody-conjugated MicroBeads and 10μl anti-THY1 antibody-conjugated MicroBeads (Miltenyi Biotech, Auburn, CA) was administered at 4°C for 20 min. with rotation. The labeled cells were filtered through 30-μm pore size mesh to remove cell aggregates, and then sorted through a separation LS column attached to a MidiMACS separator (Miltenyi Biotec). THY1+ and GFRA1+ cells were retained inside the column within the magnetic field, while unlabeled cells passed through the column and were collected as the THY1-/GFRA1- cell fraction (flow through). After washes with MACS Buffer, the LS column was removed from the magnetic field and the THY1+ and GFRA1+ cells were flushed out.

### Establishment of SSC cultures

Approximately 200,000 THY+/GFRA1+ cells were seeded into 35-mm round dishes containing irradiated Mouse Embryonic Fibroblast feeder layers (1.2 x 10^6^ MEFs per dish). Cells were maintained in optimized culture medium [55] (StemPro-34 supplemented with 1% FBS, 10 μg/mL GDNF, 10 ng/mL bFGF, 20 ng/mL EGF, 1,000 units/mL ESGRO/LIF), fed with new media every other day. SSC colonies were visualized using a Leica DM-IRB Inverted Research Microscope.

### RNA isolation and qRT-PCR analysis

Total RNA was extracted from MACS-separated cells using the RNeasy Micro Kit (Qiagen, Valencia, CA) following the manufacturer's protocol. RNA samples were treated with RNase-free DNase I (Qiagen) on-column to remove genomic DNA. Yield and quality of RNA samples were determined using the NanoDrop 2000 Spectrophotometer (ThermoScientific, Wilmington, DE). Total RNA was reverse transcribed into cDNA using random hexamer primers (Life Technologies, Grand Island, NY). For qRT-PCR, cDNA was added to 2x Power SYBR^®^ Green PCR Master Mix (Applied Biosystems, Foster City, CA) with specific oligonucleotide primer sets for the genes of interest (listed in Supplemental Table 2). Samples from three biological replicates were run in triplicate on an Applied Biosystems 7500 Real-Time PCR System using SYBR^®^ Green dye for read-out and ROX™ dye as an internal reference. Each PCR reaction contained approximately 5-10 ng of cDNA, 1x Power SYBR® Green PCR Master Mix, and 500 nM of each forward and reverse primer for the desired gene. *Gapdh* was used as an endogenous control. The threshold cycle (C_T_), indicating the relative abundance of a particular transcript, was calculated for each reaction by the system software. Quantification of the fold change in gene expression was determined by using the formula 2^−^^DD^^CT^, in which DDC_T_ = [(C_T_ of gene of interest - C_T_ of *Gapdh*)_A_ - (C_T_ of gene of interest - C_T_ of *Gapdh*)_B_]. Fold change in transcript levels was plotted using Prism 5 software (GraphPad, La Jolla, CA). Values plotted are mean +/− SEM. Statistical analysis was performed using Prism 5, employing Student's t-test or ANOVA; *p<0.05; **p<0.01; ***p<0.001.

### Gene expression microarrays

Total RNA samples were shipped on dry ice to the laboratories at Miltenyi Biotec (Auburn, CA), where they were quality-checked prior to processing with the Agilent 2100 Bioanalyzer platform (Agilent Technologies, Santa Clara, CA). Linear T7-based amplification was performed using 25 ng of each RNA sample. Amplification and Cy3-/Cy5-labeling was performed using the Agilent Low Input Quick Amp Labeling Kit (Agilent Technologies) following the manufacturer's protocol. Yields of cRNA and the dye-incorporation rate were measured using the NanoDrop 2000 Spectrophotometer (ThermoScientific). Hybridization was performed according to the Agilent 60-mer oligo microarray processing protocol using the Agilent Gene Expression Hybridization Kit (Agilent Technologies). The Cy3- and Cy5-labeled cRNAs were combined and hybridized overnight (17 hours at 65°C) onto the Agilent Whole Mouse Genome oligo microarrays 4x44K using a hybridization chamber and oven. Fluorescence signals of the hybridized oligo microarrays were detected using Agilent's DNA microarray scanner (Agilent Technologies). Agilent Feature Extraction (AFE) software was used to process the microarray image files. AFE determines feature intensities and ratios (including background subtraction and normalization), rejects outliers and calculates statistical confidences (p-values). For determining differential gene expression, AFE-derived output data files were further analyzed using the Rosetta Resolver^®^ gene expression data analysis system (Rosetta Biosoftware, Cambridge, MA). This software was used to generate a double-log scatter plot to visualize the signal intensities of all oligo probes (Figure 3A). AFE-derived output data files contained the gene lists with complete raw data sets.

### SSC transcriptome data analysis

The unsupervised clustering method AutoSOME was used to assemble our gene expression data into distinct clusters for subsequent analysis [31]. We used Euclidean distance as a user-defined parameter, scaling our data sets in log2 (establishing a relative value range from 0.0 to 20.0) and employing unit variance normalization, median centering of genes (to eliminate amplitude shifts), and normalizing genes such that the sum of squares of each row/column from our data sets=1. All gene identifiers were collapsed into a non-redundant set by averaging expression values for genes represented by more than one probe. Probes without corresponding gene symbols were not analyzed. The AutoSOME output effectively generated clusters containing gene products assembled according to closely related and interconnected expression patterns. For this study, we focused on the cluster containing *Gfra1*.

To further analyze the SSC transcriptome, we used Ingenuity Pathways Analysis^®^ (Ingenuity Systems, Redwood City, CA) to generate networks by uploading our data sets into the application. Each gene identifier was mapped to its corresponding gene object in the Ingenuity Pathways Knowledge Base (IPKB). A cutoff value of 2.00 was implemented to identify genes whose expression was significantly differentially regulated, overlaying them onto a global molecular network using information from the IPKB. Networks were algorithmically generated based on their connectivity, generating graphical representations of the molecular relationships among gene products (representing nodes) by depicting them as solid (direct) or dashed (indirect) lines. All lines are supported by at least one reference from the literature or from canonical information stored in the IPKB. Mouse, rat, and human orthologs of genes are stored as separate objects in the IPKB, but are represented as single nodes in the network. Nodes are displayed using various shapes that represent the functional class of the gene product (i.e. parallelogram = enzyme). Canonical pathway analysis utilizes well characterized metabolic and cell signaling pathways that are generated prior to data input and on which identified gene products are overlaid.

The gene expression microarray data in this study are available in the public repository Gene Expression Omnibus; accession # GSE37062.

## SUPPLEMENTAL TABLES

Supplemental Table 1 Selected differentially expressed genes from the oligo microarray (rapamycin-exposed SSCs vs controls)

Supplemental Table 2 Canonical pathways identified by Ingenuity Pathway Analysis
