# Supplementary material for: Rapamycin increases oxidative stress response gene expression in adult stem cells
Source: Aging (Albany NY). 2012 Apr 18;4(4):279–89. doi: 10.18632/aging.100451 (PMC3371763; doi:10.18632/aging.100451)
Supplement: Supplemental Table 2 — Canonical pathways identified by Ingenuity Pathway Analysis [file aging-04-279-s002.doc]

**Supplemental Table 2.** Oligonucleotide primers used for quantitative real time RT-PCR

Transcript Primer Set Reference

| *Plzf* | F: 5’-ATGGACTTCAGCACCTACGG-3’ | Payne et al., 2010 |
| --- | --- | --- |
|  | R: 5’-TGCATTCTCAGTCGCAAACT-3’ |  |
| *Oct4* | F: 5’-CAGCCAGACCCACCATCTGTC-3’ | Toyoda et al., 2009 |
|  | R: 5’-GTCTCCGATTTGCATATCTCCTG-3’ |  |
| *Lin28b* | F: 5’-GGTGGACGTCTTTGTGCACCAGAG-3’ | Payne et al., 2010 |
|  | R: 5’-CGCTCACTCCCAATACAGAACACAC-3’ |  |
| *Gfra1* | F: 5’-CTAGCCACTCTGTACTTCGT-3’ | Takayama et al., 2005 |
|  | R: 5’-GCTTGCAGCGGCAGTTGTAGA-3’ |  |
| *Ret* | F: 5’-CCAGCATCTCTATGGCGTCT-3’ | Vohra et al., 2006 |
|  | R: 5’-GCGGATCCAGTCATTCTCAT-3’ |  |
| *Nanos2* | F: 5’-GACCAGGCTCATACACTCAAG-3’ | Geyer et al., 2011 |
|  | R: 5’-GGAGGGTGTGGGTTGTG-3’ |  |
| *Sod1* | F: 5’-GACAAACCTGAGCCCTAAG-3’ | Tabuchi et al., 2011 |
|  | R: 5’-CGACCTTGCTCCTTATTG-3’ |  |
| *Gsr* | F: 5’-GCCTTTACCCCGATGTATCACGCTGTG-3’ | Jurado et al., 2003 |
|  | R: 5’-TGTGAATGCCAACCACCTTTTCCTCTTTG-3’ |  |
| *Alad* | F: 5’-CCCTGAAGACTCGGGCCTA-3’ | unpublished |
|  | R: 5’-CAATTACAAGCGAAATGAGAGCC-3’ |  |
| *Foxo1* | F: 5’-AACCAGCTCAAATGCTAGTACCATC-3’ | Kim et al., 2009 |
|  | R: 5’-CAGAAGGTTCTCCATGTTTTTCTGGA-3’ |  |
| *Erbb2* | F: 5’-GTAAGAGGCTTCTCGAAGCGCCCG-3’ | unpublished |
|  | R: 5’-CGAGGAAGCGCTGCCGCTTGTTTAT-3’ |  |
| *Wnt3a* | F: 5’-TGCTGTGTGCAGGAGAGATG-3’ | unpublished |
|  | R: 5’-TCAGGGGTTATTCGCCATAG-3’ |  |
| *Tgfbr1* | F: 5’-GAAGTGGAGCCATAGTAATGCC-3’ | unpublished |
|  | R: 5’-TCCAAGTGGAAAAATACACCG-3’ |  |
| *Stat4* | F: 5’-TGGCAACAATTCTGCTTCAAAAC-3’ | Zaheer et al., 2007 |
|  | R: 5’-GAGGTCCCTGGATAGGCATGT-3’ |  |
| *Tnf* | F: 5’-GCCGATGGGTTGTACCTTGT-3’ | Haddad and Belosevic, 2009 |
|  | R: 5’-GTGGGTGAGGAGCACGTAGTC-3’ |  |
| *Gsc* | F: 5’-CCCGTGGGCCCGGCTTGCTG-3’ | Nakaya et al., 2008 |
|  | R: 5’-CCGAGCCAAGTGGAGACGACAG-3’ |  |
| *Meox2* | F: 5’-GAGGACAGCCGCGATAGTG-3’ | Valcourt et al., 2007 |
|  | R: 5’-AGAATGGAGCTGGTCTTTGTAGGT-3’ |  |
| *Gapdh* | F: 5’-TGACGTGCCGCCTGGAGAAA-3’ | Mamo et al., 2007 |
|  | R: 5’-AGTGTAGCCCAAGATGCCCTTCAG-3’ |  |

**Supplemental References**

**1.** Geyer CB, Saba R, Kato Y, Anderson AJ, Chappell VK, Saga Y, Eddy EM. Rhox13 is translated in premeiotic germ cells in male and female mice and is regulated by nanos2 in the male. Biol Reprod. 2011. doi: 10.1095/biolreprod.111.094938.

**2.** Haddad G, Belosevic M. Transferrin-derived synthetic peptide induces highly conserved pro-inflammatory responses of macrophages. Molecular immunology. 2009;46(4):576-586.

**3.** Jurado J, Prieto-Alamo MJ, Madrid-Risquez J, Pueyo C. Absolute gene expression patterns of thioredoxin and glutaredoxin redox systems in mouse. The Journal of biological chemistry. 2003;278(46):45546-45554.

**4.** Kim DS, Itoh E, Iida K, Thorner MO. Growth hormone increases mrna levels of ppardelta and foxo1 in skeletal muscle of growth hormone deficient lit/lit mice. Endocrine journal. 2009;56(1):141-147.

**5.** Mamo S, Gal AB, Bodo S, Dinnyes A. Quantitative evaluation and selection of reference genes in mouse oocytes and embryos cultured in vivo and in vitro. BMC developmental biology. 2007;7:14.

**6.** Nakaya K, Murakami M, Funaba M. Regulatory expression of brachyury and goosecoid in p19 embryonal carcinoma cells. Journal of cellular biochemistry. 2008;105(3):801-813.

**7.** Payne CJ, Gallagher SJ, Foreman O, Dannenberg JH, Depinho RA, Braun RE. Sin3a is required by sertoli cells to establish a niche for undifferentiated spermatogonia, germ cell tumors, and spermatid elongation. Stem Cells. 2010;28(8):1424-1434.

**8.** Tabuchi K, Hoshino T, Hirose Y, Hayashi K, Nishimura B, Nakayama M, Hara A. Age-related hearing loss and expression of antioxidant enzymes in bdf1 mice. Acta oto-laryngologica. 2011;131(10):1020-1024.

**9.** Takayama T, Mishima T, Mori M, Jin H, Tsukamoto H, Takahashi K, Takizawa T, Kinoshita K, Suzuki M, Sato I, Matsubara S, Araki Y, Takizawa T. Sexually dimorphic expression of the novel germ cell antigen tex101 during mouse gonad development. Biol Reprod. 2005;72(6):1315-1323.

**10.** Toyoda S, Miyazaki T, Miyazaki S, Yoshimura T, Yamamoto M, Tashiro F, Yamato E, Miyazaki J. Sohlh2 affects differentiation of kit positive oocytes and spermatogonia. Developmental biology. 2009;325(1):238-248.

**11.** Valcourt U, Thuault S, Pardali K, Heldin CH, Moustakas A. Functional role of meox2 during the epithelial cytostatic response to tgf-beta. Molecular oncology. 2007;1(1):55-71.

**12.** Vohra BP, Tsuji K, Nagashimada M, Uesaka T, Wind D, Fu M, Armon J, Enomoto H, Heuckeroth RO. Differential gene expression and functional analysis implicate novel mechanisms in enteric nervous system precursor migration and neuritogenesis. Developmental biology. 2006;298(1):259-271.

**13.** Zaheer S, Wu Y, Bassett J, Yang B, Zaheer A. Glia maturation factor regulation of stat expression: A novel mechanism in experimental autoimmune encephalomyelitis. Neurochemical research. 2007;32(12):2123-2131.
